# Fillers: Contraindications, Side Effects and Precautions

**DOI:** 10.4103/0974-2077.63222

**Published:** 2010

**Authors:** Philippe Lafaille, Anthony Benedetto

**Affiliations:** *Dermatologic Surgi Center, 1200 Locust Street, Philadelphia, PA 19107, USA*

**Keywords:** Contraindications, fillers, side effects

## Abstract

Fillers are generally considered safe. However side effects may happen and hence a practicing dermatologist need to be aware of such side effects, contraindicatons and precaution to be adopted while using fillers.

## INTRODUCTION

Soft tissue fillers are increasingly used for aesthetic purposes for the rejuvenation of the face and selected areas of the body. Various materials are now available that may have different side effect profiles. Although unwanted reactions are rare, the clinician must be aware of such risks, to recognize them and treat them properly, and also to be able to prevent them with the use of proper techniques.

## COMMON COMPLICATIONS

The common complications encountered with all types of soft tissue fillers can be categorized according to different criteria:

1. The time of onset: Early versus delayed reaction [Table 1].
2. Aesthetic relevance: minor versus major.
3. Causality of the adverse reaction:
  - injection site reactions;
  - adverse effects resulting from an improper injection technique;
  - allergies and hypersensitivity reactions and
  - vascular mediated events.

**Table 1:** Side effects categorized according to the time of onset[17]

Early (days to weeks)	Delayed (weeks to years)
Injection site reaction	Granulomatous inflammation/foreign body reaction
Swelling	
Redness	Nodules, erythematous or not
Bruising/ecchymosis	
Pain	
Itching	
Infections	
Allergic reaction/hypersensitivity	Migration of the implanted filler
Inflammation	
Solid nodules	
Lumps caused by misdistribution	Scarring
Tissue necrosis	Asymmetry
Embolism with blindness	

This article discusses the side effects as per the causality.

### Injection site reactions

Injection site reactions from the needle puncture of the skin can result in swelling, bruising, redness, pain, itching, and infections. A certain amount of swelling and bruising is expected and usually lasts no more than a few days. The swelling and bruising can be minimised by applying firm pressure and ice-packs before, after or at both times during a treatment session. Use of unnecessary anti-coagulant medications or products, if any, should be stopped. A rare form of recurrent and intermittent swelling, occurring after the ingestion of alcohol, sunlight exposure or vigorous exercise, lasting for years after the injection has been reported.[1]

Infection is a rare event, and it can present as a single or multiple erythematous and fluctuant nodules that are best treated with antibiotics active against frequent skin bacteria. Some authors have hypothesized that inflammatory nodules may be caused by low-grade infection of bacteria (like *Staphylococcus epidermidis* or *Propionibacterium acnes*) maintained within a biofilm (a mixture of bacteria, nutrients and waste products) around the filler.[2,3] Filler injections should not be performed if there is an adjacent site of infection e.g. intraoral, mucosal or dental infection or herpes labialis for lip injection. There is no evidence-based data to support the idea that fillers play a triggering role in recurrent herpes infection and therefore there is no rationale in using an antiherpes prophylaxis regimen with every patient. However, patients who have had an history of developing cold sore after a filler injection could benefit from it.[2,3]

### Inappropriate technique

**Figure 1:**
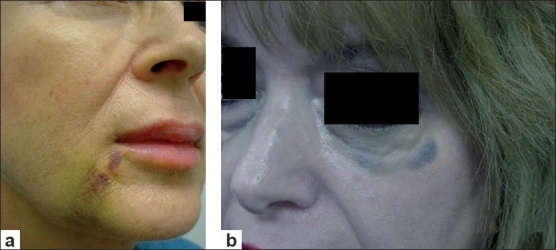
(a) Nasolabial fold ecchymosis 5 days after Radiesse injection; (b) ecchymosis of the lower eyelid 7 days after hyaluronic acid injection

**Figure 2:**
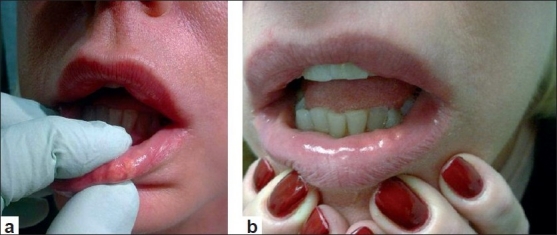
(a) and (b) Nodules in the lower lip 1 month after hyaluronic acid injection

**Figure 3:**
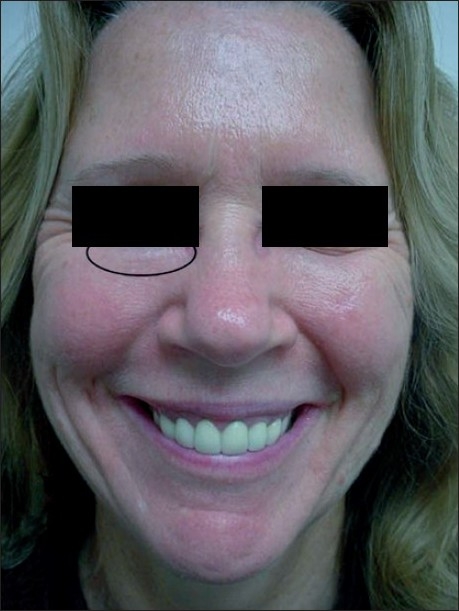
Nodule in the lower eyelid after hyaluronic acid injection with the Tyndall phenomenon

Adverse effects like palpable implants, visible implants, over- or undercorrection usually result from an improper injection technique.[3] Depending on the type of filler used (especially with hyaluronic acid fillers), injections applied too superficially can lead to small nodule formation or bluish discolouration under the skin [Figure 1a and b and Figure 2a and b]. This bluish discolouration [Figure 3] is due to the Tyndall phenomenon and is also caused by the presence of traces of hemosiderin due to post-injection intradermic bleeding.[4] Small nodules can be treated with local massage, aspiration or incision and drainage of the product. Hyaluronidase can be used to dissolve a nodule or a focus of overcorrection in the case of hyaluronic acid-based fillers. However, a preliminary skin test is necessary to rule out an allergic reaction to hyaluronidase.[5]

If Artecoll™ (collagen and polymethylmethacrylate) is injected too superficially, it can lead to persistent itchiness, redness and even hypertrophic scarring, that may need to be reduced by local corticosteroid injections.[6] Superficial injection of Radiesse™ (calcium hydroxylapatite) can lead to small whitish nodules on the surface of the skin, which may be punctured and their contents expressed. Superficial silicone injections can induce fibrosis and foreign body granulomas called siliconomas resulting in nodules, beading and textural changes.[7] Siliconomas and scarring, can occur after a variable delay after the silicone injections, ranging from 3 weeks to a decade.[8]

Accidental intramuscular injection of a synthetic filler other than hyaluronic acid and collagen should be avoided since the repetitive contraction of the muscle will often dislocate the filler and create unwanted lumps of material and cause it to migrate to distant sites.[9] The use of Radiesse in the lips is usually avoided for this reason, because the migration of the filler caused by the repetitive contraction of the orbicularis oris can cause superficial nodules of the filler to occur.[9]

### Allergies and hypersensitivities

Different types of reactions can occur depending upon the material used and these are discussed here.

#### Collagens

Bovine collagen can be immunogenic and cause a foreign body granulomatous reaction at the rate of 1.3%.[6] Erythematous, indurated reactions or subcutaneous papules have been reported as early as 10 days after the first injection.[10] About 1–3% of patients with one negative skin test may still develop a reaction to the filler.[11] Consequently, two pre-treatment skin tests are recommended 3–4 weeks apart to make sure that the patient is not allergic to bovine collagen. A systemic hypersensitivity reaction with fever, malaise and urticaria, occurring 2–3 days after the injection has also been described with Zyderm™ and Zyplast™. It responds well to a short-term course of oral glucocorticoid therapy but treatment is not always necessary as it may be self-limiting.[12]

#### Hyaluronic acid fillers

Granulomatous foreign body reaction can develop months to years after the injection. This reaction may be asymptomatic or have associated erythema and swelling. Histologically, a collection of macrophages with some multinucleated giant cells and surrounding lymphocytes are seen.[6] Persistent granulomatous foreign body reactions can be treated with intralesional corticosteroid injections. The usual dosage would be 5–10 mg/cc depending on the severity of the reaction and should be repeated if necessary 4–6 weeks later. Some authors have advocated the use of hyalorunidase[5,13] hoping that the break-down of the product would stop the foreign body reaction. One angioedema-type reaction without airway involvement was reported after the injection of hyaluronic acid (Restylane) in a lip.[14]

#### Poly-L-lactic acid

In the US clinical studies, skin nodules have been described in HIV-infected individuals and occur on average 3–4 months post-treatment, but can occur as early as 1 month after the injections. The nodules are typically palpable, asymptomatic and non-visible and occur in up to 13% of patients in the American HIV lipoatrophy studies.[15] The exact nature and mechanism for these nodules is not known but experience has shown that injections should be made below the dermis to avoid their detection.

### Vascular mediated event

Skin necrosis by compression or obstruction of a blood vessel is a rare complication. The site the most at risk for this complication is the glabella since this region is supplied by the suptratrochlear artery, which does not have a strong collateral circulation.[6] To avoid this complication, it is better to use a less dense filler (Restylane Touch, Zyderm 1, Cosmoderm I) in this region and inject it very superficially and slowly, while constantly moving the needle. Other fillers including autologous fat have been reported to cause glabellar necrosis. Injecting low volumes over two or three sessions instead of using a high volume in one session may also help in preventing this complication.[6] Retinal embolism with the intravascular injection of the supratrochlear artery (connected to the ophthalmic artery) has also been described.[16] Withdrawing with the needle before injecting and injecting the filler in a constant back and forth movement could help prevent this complication.[9]

## CONTRAINDICATIONS

The major contraindications to the use of a filler are as follows: active infection near the site of injection, a known allergy/hypersensitivity to the material or to the lidocaine mixed in the syringe of the filler (Zyderm, Zyplast, Cosmoderm, Cosmoplast and certain hyaluronic acid fillers and Artefill) and glabellar skin necrosis with injected Zyplast [Table 2]. No causal relationship has been established between the use of filler and autoimmune diseases like dermatomyositis/polymyositis, lupus erythematosus, rheumatoid arthritis or scleroderma. Their use is therefore not contraindicated in patients suffering from those diseases. Immunosuppression has not been found to increase the risk of complications linked to the use of fillers other than poly-L-lactic acid. Table 2 lists conditions where a filler can be used safely and where it cannot be used.

**Table 2:** Use of fillers

Contraindication	Not a contraindication
Active infection	Autoimmune disease
	Dermatomyositis/polymyositis
	Lupus erythematosus
	Rheumatoid arthritis
	Scleroderma
Allergy/hypersensitivity to the filler	Immunosuppression
Glabellar necrosis	
Allergy to lidocaine (collagen, hyaluronic acids, Artefill)	

## PRECAUTIONS FOR AVOIDING COMPLICATIONS

1. The first step in minimizing unwanted results is to begin by injecting temporary fillers. This will enable both the patient and the treating physician to evaluate the cosmetic results and decide whether or not a more permanent filler would be desirable.[3]
2. Swelling and bruising can be minimised by avoiding the use of anticoagulant medication or over-the-counter products and applying ice-packs before and after a treatment along with gentle but firm pressure after a treatment.
3. A thorough understanding of the different recommended depths for injecting different products is also mandatory to avoid the reactions associated with injections placed too superficially. As a general rule, non-permanent, absorbable fillers can be injected more superficially and the more permanent fillers need to be injected more deeply.
4. Exaggerated and repeated movements should be minimised during the first 3 days after a treatment to minimise product migration/displacement.[9].
5. Some anatomical locations and types of scars are more susceptible to develop unwanted reactions and results. For example, ridging or beading on the sides of a rhytide occurs more frequently around the horizontal lines of the forehead and the vertical lines around the mouth. Areas with thin skin, such as the eyelids and the area around the eyes, should be avoided as the injected filler material can be easily displaced underneath the thin and distensible skin. Ridging or beading can therefore occur at the area of the crow's feet. However, some fillers such as Cosmoderm, Zyderm I, Restylane Fine Line are suitable for injection in this area and may yield better results. The glabella is more at risk for skin necrosis and ulceration after injection with a dense filler like Zyplast or autologous fat. There have been reports of the induction of amaurosis possibly due to thrombus formation in the retinal artery after an injection of bovine collagen.[18] Narrow, deep and depressed scars as a result of varicella and acne (especially the ‘ice-pick’-type scars) are susceptible to a phenomenon called ‘doughnutting’ where the periphery of the scar elevates with the filler above the depressed or retracted centre of the scar.[3]
6. As already stated, bovine-derived collagen products must be skin tested before treatment.

## MANAGING COMPLICATIONS

If severe, unremitting swelling (more often seen with hyaluronic acid-based fillers or Radiesse) or allergic reactions occur, they can be managed with oral corticosteroids and antihistamines.

If ridging or beading occurs with a non-permanent implant, it will usually resolve with time. If considered necessary, the filler can be extracted within the first few days of a treatment by the technique of incision and drainage of the clumped accumulation of the filler. Permanent filler implants can be managed with intralesional corticosteroids, surgical excision, dermabrasion or CO_2_ laser resurfacing.[3] Small upper lip nodules resulting from a treatment of Radiesse may diminish by vigorous lip massage, incision and drainage, intralesional steroid or surgical excision.[19]

If prolonged blanching and pain occurs while injecting the filler, there is a possibility that cutaneous arteriolar occlusion has occurred. The immediate administration of heat, massage of the area and application of the nitroglycerin paste should be performed while the patient is still in the office and at home by the patient until the symptoms subside. If a hyaluronic acid-based filler was used, hyaluronidase can be injected in the blanched, painful area and around the vessels involved and in order to disrupt the product and decompress the vessel in the hypoxic area.

Granulomas and foreign body reactions can be managed with either intralesional or systemic corticosteroids, or antibiotics if the nodules are persistently inflammatory.[1,2] A report of the use of imiquimod to treat silicone-induced granulomas of the lip has been published.[20]

## CONCLUSION

Many strategies exist to reduce the risks of filler complications. A good working knowledge of the different side effect profiles of each available product is mandatory. Although unwanted reactions are rare when a proper injection technique is used, patients should be aware that unforeseen adverse sequelae can always occur.
